# The Second Selectivity of Taxanes to Malignant Cells --- Nuclear Envelope Malleability

**DOI:** 10.7150/jca.104809

**Published:** 2025-01-01

**Authors:** Xiang-Xi Xu, Elizabeth R. Smith

**Affiliations:** 1 Sylvester Comprehensive Cancer Center, University of Miami Miller School of Medicine, Miami, FL 33136, USA.; 2 Department of Radiation Oncology, University of Miami Miller School of Medicine, Miami, FL 33136, USA.; 3 Department of Obstetrics, Gynecology and Reproductive Sciences, University of Miami Miller School of Medicine, Miami, FL 33136, USA.

**Keywords:** chemotherapy, taxanes, Taxol, paclitaxel, microtubules, mitosis, proliferation, nuclear envelope, micronuclei, membrane rupture, specificity/selectivity

We wish to suggest a cellular mechanism expanding to the understanding of the cancer selectivity/specificity of taxanes, a group of commonly used cancer drugs by the action of microtubule stabilization.

The current frontline treatment of several major solid tumors is a taxane-based chemotherapy that was formulated nearly forty years ago, though with refinement over time. Taxanes work through a mechanism of microtubule stabilization 1-4. Currently, several principal taxanes, such as Taxol/paclitaxel, Taxotere/docetaxel, and Jevtana/cabazitaxel, are used as a frontline regimen in combination with other drugs (often platinum agents), as well as second line drugs for recurrent cancer. Taxanes are highly active in many major solid tumors, and especially useful for treating malignant and metastatic cancers, including those of breast, lung, prostate, ovarian, head and neck, and cervical carcinomas, with mostly tolerable side effects 5-9. Nearly all cancer patients with these tumor types will likely be treated with taxanes at one point in the course of managing their diseases.

The initial discovery of the activity of paclitaxel/taxol (the first taxane) in stabilization of cellular microtubules and consequential mitotic arrest of cancer cells propelled the enthusiasm for the development of paclitaxel as a cancer drug 10,11. Commonly, paclitaxel's anti-cancer activity (and also of all other taxanes) is thought to be conferred by its binding to and stabilizing cellular microtubules, which interferes with mitosis and leads to cell growth arrest 1-3,12 and subsequent mitotic slippage and mitotic catastrophe 13,14. Thus, taxanes are considered mitotic inhibitors. The major side effects of taxanes, myelosuppression and alopecia, are consistent with the idea that taxanes target mitotic cells, such as the rapidly renewing hematopoietic cells and the continuously proliferating hair matrix cells 15,16. However, the molecular mechanism leading to cell death has not been clearly deciphered 17-20.

Overtime, some skepticisms persisted with regards to the idea that blocking mitosis is the sole mechanism of action for taxanes on cancer cells 17,21-24. For one, taxane cell killing activity does not correlate with the rate/index of mitosis or proliferation of the treated tumors 25, an observation known as the mitotic paradox 26. The lack of clinical activity of other mitotic inhibitors also casts doubt on mitotic inhibition as the sole mechanism of taxanes 22,27.

In laboratory studies using cancer cells, upon treatment with taxanes, the nuclei of cancer cells fragment into multiple micronuclei, a process known as micronucleation 20,28-30. The taxane-induced generation of multiple micronuclei occurs in both mitotic 13,29,31 and also in non-mitotic cells 32. A mechanism was proposed that the paclitaxel-induced rigid microtubule bundles physically pull the nuclear envelope through the LINC (Linker of nucleoskeleton and cytoskeleton) bridges and break the nucleus off to form micronuclei 33. Additionally, the generated multiple micronuclei have a weakened nuclear envelope and membrane, for which the stretching of surface to form multiple spheres of micronuclei from a single nucleus is one of the feasible explanations 33. The catastrophic rupture of the micronuclei consequently leads to cell death 20,28-30.

Here, nuclear envelope malleability/fragility refers to softened nuclear membranes and envelope, presented as nuclear morphology deformation that is a common characteristic of cancer cells. Laboratory experiments using cultured cells led to a conclusion that nuclear envelope malleability/fragility, which is modulated and controlled by overexpression or suppression of nuclear envelope lamina Lamin A/C, determines sensitivity of cells to paclitaxel-induced micronucleation and cell death 32-34. This new mechanistic understanding provides an explanation for the non-mitotic action of taxanes, by inducing micronucleation as a result of rigid microtubule bundles pulling the malleable and fragile nuclear envelope of cancer cells 33,34.

This new understanding of taxane mechanism beyond mitotic inhibition prompted us to reassess the reason(s) why taxanes are more successful in clinic than expected, and how taxanes are more toxic to cancer than to normal host cells in addition to inhibiting the cell proliferation rate 35. Further appraisal of the experimental conclusions leads to the recognition of the susceptibility of cancer nuclear envelopes subjected to fragmentation by the drug-induced rigid microtubule bundles as a second selectivity/specificity of taxanes (**Fig. 1**). Nuclear envelope malleability/fragility is often determined by nuclear envelope lamina proteins 33-36, and particularly Lamin A/C level for cancer cells 37,38. Cancer cells generally have a reduced Lamin A/C protein level 37-41, which has been also suggested to be a cause of aneuploidy through nuclear budding 42. Another consequence of reduced Lamin A/C protein in cancer cells and the property of nuclear envelope malleability is the morphological deformation of the cancer nucleus 37,43, which is the basis in diagnosis of malignant cells in the PAP smear test 38. Thus, cancer cells with deformed nuclear morphology and massive aneuploidy, which are commonly present in malignant and metastatic carcinomas, can be predicted to be sensitive and responsive to taxane treatment. In contrast, benign cells with a sturdy nuclear envelope presenting a smooth and oval shaped morphology are more resistant to taxane-induced micronucleation and rupture (**Fig. 1**).

In summary, we suggest that nuclear envelope malleability/fragility that is often caused by a reduced nuclear envelope structural protein (Lamin A/C) which is the second selectivity/specificity of cancer cells to taxanes (**Fig. 1**). These two properties of cancer cells, high proliferation rate and malleable nuclear envelope, may provide two aspects of specificity and selectivity to taxanes and contribute to the surprising success of taxanes in cancer treatment over the last four decades. The recognition of a second selectivity/specificity likely will prompt oncologists to revisit the rationale for optimal use of taxanes in cancer management. Subsequent new understanding may enable additional rational combinations of taxanes in oncology, and may inspire new strategies to more efficiently counter cancer.

## Figures and Tables

**Figure 1:**
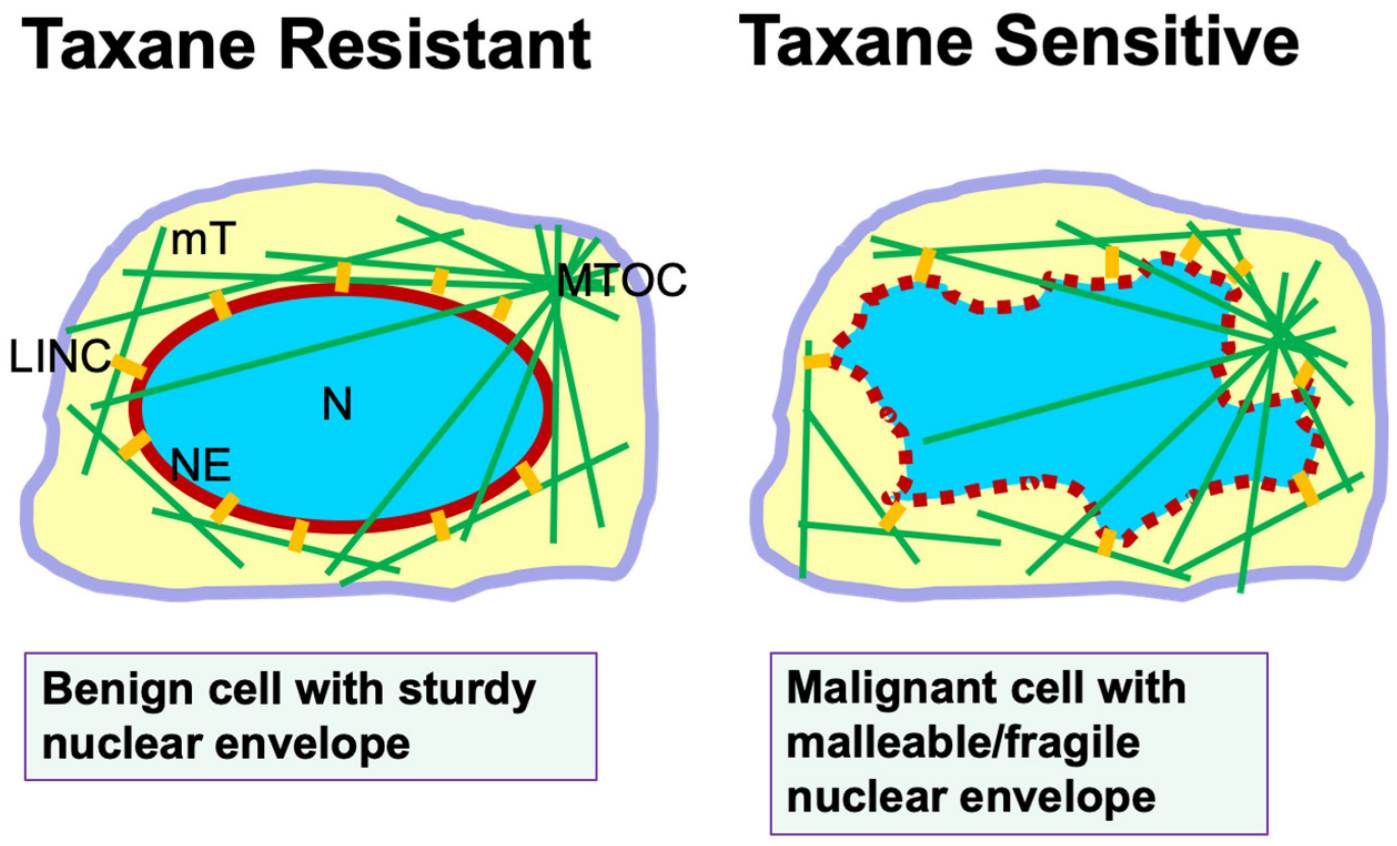
** Nuclear envelope malleability/fragility is a predictor of taxane sensitivity.** Illustration shows a benign cell with sturdy nuclear envelope and exhibiting a smooth and oval-shaped morphology. Microtubules extend out from the microtubule organizing center (MTOC). The nuclear envelope is connected to the microtubule cytoskeleton through the LINC bridges (short yellow lines). In a malignant cell, the malleable nuclear envelope (depicted by a dotted red line) is disturbed by physical pulling from microtubules through the LINC bridges. In the presence of taxanes, cellular microtubules are stabilized and bundled, and the rigid filaments pull apart the fragile nuclear envelope to form multiple micronuclei, leading to cell death. Thus, the property of a malleable/fragile nuclear envelope in cancer cells provides a second specificity/selectivity for taxane killing though micronucleation and nuclear envelope rupture. Benign cells with a sturdy nuclear envelope are more resistant to taxane-induced micronucleation, and are taxane resistant. **Abbreviations: mT,** microtubules;** LINC,** linker of nuclear and cytoplasmic skeleton;** N,** nucleus;** MTOC**, microtubule organizing center;** NE,** nuclear envelope.

## References

[1] Horwitz SB (1994). Taxol (paclitaxel): mechanisms of action. Ann Oncol.

[2] Jordan MA, Toso RJ, Thrower D, Wilson L (1993). Mechanism of mitotic block and inhibition of cell proliferation by taxol at low concentrations. Proc Natl Acad Sci. USA.

[3] Jordan MA, Wilson L (2004). Microtubules as a target for anticancer drugs. Nat Rev Cancer.

[4] Gallego-Jara J, Lozano-Terol G, Sola-Martínez RA, Cánovas-Díaz M, de Diego Puente T (2020). A compressive review about taxol: History and future challenges. Molecules.

[5] Rowinsky EK, Donehower RC (1995). Paclitaxel (taxol). N Engl J Med.

[6] Friedrich M, Diesing D, Villena-Heinsen C, Felberbaum R, Kolberg HC, Diedrich K (2004). Taxanes in the first-line chemotherapy of metastatic breast cancer: review. Eur J Gynaecol Oncol.

[7] Baird RD, Tan DS, Kaye SB (2010). Weekly paclitaxel in the treatment of recurrent ovarian cancer. Nat Rev Clin Oncol.

[8] Bookman MA (2016). Optimal primary therapy of ovarian cancer. Ann Oncol.

[9] Mosca L, Ilari A, Fazi F, Assaraf YG, Colotti G (2021). Taxanes in cancer treatment: Activity, chemoresistance and its overcoming. Drug Resist Updat.

[10] Schiff PB, Fant J, Horwitz SB (1979). Promotion of microtubule assembly in vitro by taxol. Nature.

[11] Schiff PB, Horwitz SB (1980). Taxol stabilizes microtubules in mouse fibroblast cells. Proc Natl Acad Sci U S A.

[12] Jordan MA (2002). Mechanism of action of antitumor drugs that interact with microtubules and tubulin. Curr Med Chem Anticancer Agents.

[13] Weaver BA (2014). How Taxol/paclitaxel kills cancer cells. Mol Biol Cell.

[14] Morse DL, Gray H, Payne CM, Gillies RJ (2005). Docetaxel induces cell death through mitotic catastrophe in human breast cancer cells. Mol Cancer Ther.

[15] Rowinsky EK, Eisenhauer EA, Chaudhry V, Arbuck SG, Donehower RC (1993). Clinical toxicities encountered with paclitaxel (Taxol). Semin Oncol.

[16] Visconti R, Grieco D (2017). Fighting tubulin-targeting anticancer drug toxicity and resistance. Endocr Relat Cancer.

[17] Blagosklonny MV, Fojo T (1999). Molecular effects of paclitaxel: myths and reality (a critical review). Int J Cancer.

[18] Blagosklonny MV, Robey R, Sheikh MS, Fojo T (2002). Paclitaxel-induced FasL-independent apoptosis and slow (non-apoptotic) cell death. Cancer Biol Ther.

[19] Barbuti AM, Chen ZS (2015). Paclitaxel through the ages of anticancer therapy: Exploring its role in chemoresistance and radiation therapy. Cancers (Basel).

[20] Mitchison TJ, Pineda J, Shi J, Florian S (2017). Is inflammatory micronucleation the key to a successful anti-mitotic cancer drug?. Open Biol.

[21] Komlodi-Pasztor E, Sackett D, Wilkerson J, Fojo T (2011). Mitosis is not a key target of microtubule agents in patient tumors. Nat Rev Clin Oncol.

[22] Komlodi-Pasztor E, Sackett DL, Fojo AT (2012). Inhibitors targeting mitosis: tales of how great drugs against a promising target were brought down by a flawed rationale. Clin Cancer Res.

[23] Fürst R, Vollmar AM (2013). A new perspective on old drugs: non-mitotic actions of tubulin-binding drugs play a major role in cancer treatment. Pharmazie.

[24] Field JJ, Kanakkanthara A, Miller JH (2014). Microtubule-targeting agents are clinically successful due to both mitotic and interphase impairment of microtubule function. Bioorg Med Chem.

[25] Schimming R, Mason KA, Hunter N, Weil M, Kishi K, Milas L (1999). Lack of correlation between mitotic arrest or apoptosis and antitumor effect of docetaxel. Cancer Chemother Pharmacol.

[26] Mitchison TJ (2012). The proliferation rate paradox in antimitotic chemotherapy. Mol Biol Cell.

[27] Yan VC, Butterfield HE, Poral AH, Yan MJ, Yang KL, Pham CD, Muller FL (2020). Why great mitotic inhibitors make poor cancer drugs. Trends Cancer.

[28] Panvichian R, Orth K, Day ML, Day KC, Pilat MJ, Pienta KJ (1998). Paclitaxel-associated multimininucleation is permitted by the inhibition of caspase activation: a potential early step in drug resistance. Cancer Res.

[29] Zhu Y, Zhou Y, Shi J (2014). Post-slippage multinucleation renders cytotoxic variation in anti-mitotic drugs that target the microtubules or mitotic spindle. Cell Cycle.

[30] Merlin JL, Bour-Dill C, Marchal S, Bastien L, Gramain MP (2000). Resistance to paclitaxel induces time-delayed multinucleation and DNA fragmentation into large fragments in MCF-7 human breast adenocarcinoma cells. Anti-Cancer Drugs.

[31] Zasadil LM, Andersen KA, Yeum D, Rocque GB, Wilke LG, Tevaarwerk AJ, Raines RT, Burkard ME, Weaver BA (2014). Cytotoxicity of paclitaxel in breast cancer is due to chromosome missegregation on multipolar spindles. Sci Transl Med.

[32] Smith ER, Leal J, Amaya C, Li B, Xu XX (2021). Nuclear Lamin A/C Expression Is a Key Determinant of Paclitaxel Sensitivity. Mol Cell Biol.

[33] Smith ER, Xu XX (2021). Breaking malignant nuclei as a non-mitotic mechanism of taxol/paclitaxel. J Cancer Biol.

[34] Smith ER, Wang JQ, Yang DH, Xu XX (2022). Paclitaxel resistance related to nuclear envelope structural sturdiness. Drug Resist Updat.

[35] Smith ER, Li Z, Chen Z, Xu XX (2024). Reassessing specificity/selectivity of taxane-based chemotherapy. Cancer Insight.

[36] Smith ER, Meng Y, Moore R, Tse JD, Xu AG, Xu XX (2017). Nuclear envelope structural proteins facilitate nuclear shape changes accompanying embryonic differentiation and fidelity of gene expression. BMC Cell Biol.

[37] Capo-chichi CD, Cai KQ, Smedberg J, Ganjei-Azar P, Godwin AK, Xu XX (2011). Loss of A-type lamin expression compromises nuclear envelope integrity in breast cancer. Chin J Cancer.

[38] Capo-chichi CD, Cai KQ, Simpkins F, Ganjei-Azar P, Godwin AK, Xu XX (2011). Nuclear envelope structural defects cause chromosomal numerical instability and aneuploidy in ovarian cancer. BMC Med.

[39] Smith ER, George SH, Kobetz E, Xu XX (2018). New biological research and understanding of Papanicolaou's test. Diagn Cytopathol.

[40] Moss SF, Krivosheyev V, de Souza A, Chin K, Gaetz HP, Chaudhary N, Worman HJ, Holt PR (1999). Decreased and aberrant nuclear lamin expression in gastrointestinal tract neoplasms. Gut.

[41] Foster CR, Przyborski SA, Wilson RG, Hutchison CJ (2010). Lamins as cancer biomarkers. Biochem Soc Trans.

[42] Capo-Chichi CD, Yeasky TM, Smith ER, Xu XX (2016). Nuclear envelope structural defect underlies the main cause of aneuploidy in ovarian carcinogenesis. BMC Cell Biol.

[43] Zink D, Fischer AH, Nickerson JA (2004). Nuclear structure in cancer cells. Nat Rev Cancer.

